# Population genetic structure in six sympatric and widespread aquatic plants inhabiting diverse lake environments in China

**DOI:** 10.1002/ece3.3141

**Published:** 2017-06-15

**Authors:** Qian‐Jin Cao, Fang‐Fang Mei, Ling Wang

**Affiliations:** ^1^ School of Life Sciences Central China Normal University Wuhan Hubei China

**Keywords:** bird‐mediated dispersal, environment, gene flow, hydrologic connectivity, plateau lake

## Abstract

Many aquatic plant species are distributed over large areas and diverse environments with populations interconnected by abiotic and biotic mediators. Here, we examined differences and similarities in the population genetic structure of six sympatric and widespread aquatic plant species. We sampled the aquatic species from six Chinese lakes found on plateaus, plains, and different river systems and analyzed them using inter‐simple sequence repeat (ISSR) markers. Samples originating from each lake tended to cluster together. Of the six species, only *Nymphoides peltata* and *Myriophyllum spicatum* could be divided into plateau and plain groups, once Taihu Lake individuals were excluded. Genetic similarities between populations connected by the Yangtze River were not consistently higher than unconnected populations. Populations from Taihu Lake and/or Weishanhu Lake were distant from other lake populations for all species except *Potamogeton lucens*. The Taihu and Weishanhu populations clustered for *Ceratophyllum demersum* and *Typha latifolia*. Hydrophilous *C. demersum* had the lowest gene flow (*Nm *= 0.913), whereas the entomophilous *Hydrocharis dubia* (*Nm *= 2.084) and *N. peltata* (*Nm *= 2.204) had the highest gene flow. The genetic relationships among distant populations of aquatic plants reflect the comprehensive effects of environmental selection pressure and biotic and abiotic connectivity. Differences in environmental factors between plateau and plain lakes and long distance hydrochory have limited importance on aquatic plant genetic structures. Among multiple evolutionary forces, gene flow mediated by birds may play the most important role in the formation of genetic patterns in the six species examined. For example, the close genetic relationship between Taihu Lake and Weishanhu Lake populations, each in different river systems and with different climates, may be related to the migration routes of birds. Differences in gene flow among the six aquatic plants may be attributable to different bird‐transport and the fruit traits of each species.

## 1 INTRODUCTION

Many aquatic plants are distributed over large areas and diverse environments and comprise populations interconnected by abiotic (e.g. water) and biotic mediators (e.g. birds; Barrat‐Segretain, 1996; Lacoul & Freedman, 2006; Santamaría, 2002; Wu, Yu, Li, & Xu, 2016).

Differential selection can generate adaptive population divergence (Barrett, Eckert, & Husband, 1993; Wang & Bradburd, 2014). In contrast, connectivity mediated by water and birds favors gene flow and tightens genetic relationships among populations (Chen, Li, Yin, Cheng, & Li, 2009; Chen, Xu, & Huang, 2007; Nilsson, Brown, Jansson, & Merritt, 2010; Pollux, Luteijn, Van Groenendael, & Ouborg, 2009; Wang, Song, Liu, Lu, & Li, 2010). Over evolutionary time, these opposite forces work interactively on widespread species inhabiting similar areas, but the question remains: how are patterns of population genetic structure for sympatric widespread plants similar?

China has many large lakes in diverse environments. Due to shifts in elevation, climates on the Yunnan‐Guizhou Plateau (subtropical plateau monsoon climate) and broad plains on the third step of the terrain ladder in China (Zheng, 2015) are quite different. Furthermore, on the third terrain step, large differences in latitude lead to different climates on the North China Plain (warm temperate monsoon climate) and plains of the middle and lower reaches of the Yangtze River (subtropical monsoon climate). These environmental differences may result in different selection pressures on aquatic plants, different adaptations to local environments, and genetic differentiation among species inhabiting these lakes.

However, some lakes located in different climates are connected by rivers. For example, the Yangtze River connects many lakes from western upstream plateaus to eastern midstream and downstream plains (Wang & Dou, 1998). This hydrologic connectivity may facilitate water‐ and fish‐mediated propagule dispersal and increase genetic similarity among connected lakes (Chen et al., 2007, 2009; Pollux et al., 2009; Wang et al., 2010). Although the dispersal distance of hydrochory is limited, propagules being re‐dispersed in several episodes may result in long distance dispersal (Nilsson et al., 2010). Several bird migration routes traverse northern and southern China (Li & Yang, 1997) and this may also increase gene flow among lake populations.

In this study, we sampled six aquatic plant species, which differ in life forms and pollination modes, from six lakes (two plateau lakes and four plain lakes) separated by large distances and in different climatic zones in China. Among the focal lakes, one plateau lake and three plain lakes are connected by the Yangtze River, and two of the plain lakes are located on the same avian migratory route. By comparing the genetic relationships among lake populations of different plant species, we hope to further understand environmental effects, hydrologic connectivity, and bird migration on population genetic structures in aquatic species. We hypothesize that (1) population genetic differentiation could arise from divergent environmental pressures or demographic factors related to differential population sizes and isolation of lakes, whereas (2) gene flow mediated by water, fish, and birds homogenizes populations.

## 2 MATERIALS AND METHODS

### 2.1 Site description

**Figure 1:**
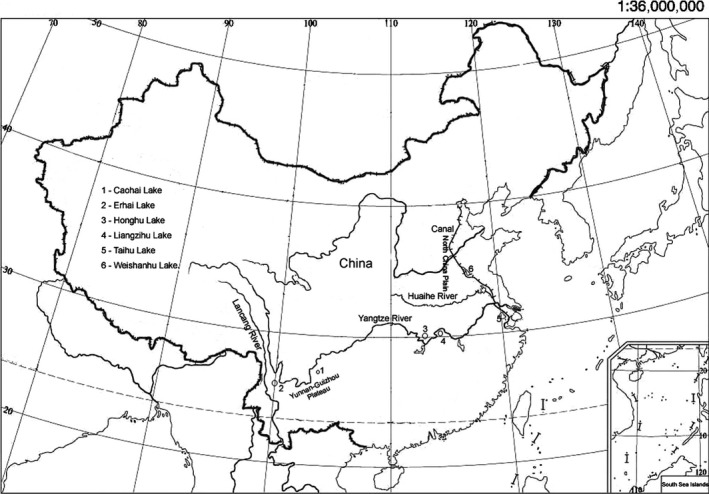
Map showing locations of the six lake populations sampled in this study

Caohai Lake and Erhai Lake are located on the Yunnan‐Guizhou Plateau in a subtropical plateau monsoon climate (Table 1, Figure 1). Among the four plain lakes, Honghu Lake, Liangzihu Lake, and Taihu Lake are located in the middle and lower reaches of the Yangtze River in a subtropical monsoon climate, and Weishanhu Lake is on the North China Plain in a warm temperate monsoon climate. Erhai Lake is part of the Lancang River system; Weishanhu Lake is within the Huaihe River system; and the other four lakes are connected by the Yangtze River.

**Table 1:** Sampling sites in this study

Location/Climate	Lake (population)	River system	Latitude (N)^a^	Longitude (E)	Elevation (m)	*T* (°C)^b^	P (mm)
Yunnan‐Guizhou Plateau/Subtropical plateau monsoon climate							
Weining, Guizhou	Caohai	Yangtze River	26°50.65′–26°51.25′	104°15.09′–104°16.23′	2152–2180	10.4	950.9
Dali, Yunnan	Erhai	Lancang River	25°37.37′–25°56.59′	100°05.90′–100°16.42′	1963–1992	15.0	1056.6
Plain of middle and lower reaches of the Yangtze River/Subtropical monsoon climate							
Honghu, Hubei	Honghu	Yangtze River	29°49.11′–29°51.27′	113°20.95′–113°23.67′	19–28	16.6	1343.3
Wuhan, Hubei	Liangzihu	Yangtze River	30°14.56′–30°16.51′	114°32.99′–114°38.52′	14–28	16.8	1263.4
Suzhou, Jiangsu	Taihu	Yangtze River	31°04.82′–31°07.88′	120°21.01′–120°23.88′	2–7	16.0	1092.0
North China Plain/Warm temperate monsoon climate							
Weishan, Shandong	Weishanhu	Huaihe River	34°39.70′–34°40.33′	117°16.60′–117°17.04′	29–42	14.2	684.0

a Latitude, longitude, and elevation were recorded when samples were collected.

b T, average annual temperature; P, average annual precipitation from Wang and Dou (1998).

### 2.2 The biology of species

**Figure 2:**
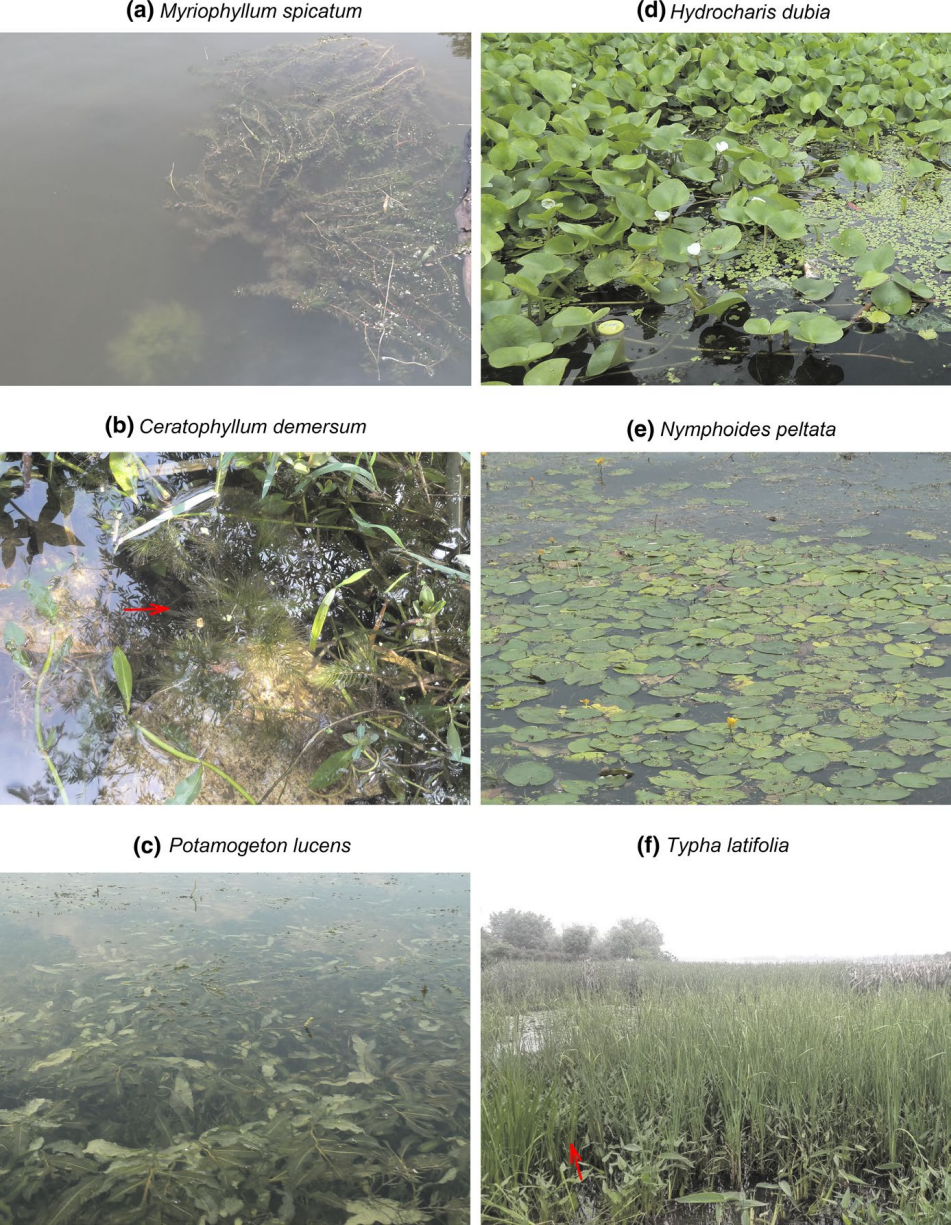
Photo of study species in wild populations. Each panel of (a) to (f) exhibits one species. The red arrows denote the focus species

The six species studied are indigenous and widely distributed in China. Three submerged (*Myriophyllum spicatum* L., *Ceratophyllum demersum* L., and *Potamogeton lucens* L.), two floating‐leaved (*Hydrocharis dubia* (Bl.) Backer and *Nymphoides peltata* (Gmel.) O. Kuntze), and one emergent species (*Typha latifolia* L.) were included (Figure 2). Of the above species, *C. demersum* and *H. dubia* are able to float freely in the water column and on the water surface, respectively, and able to be transported by water as whole plant diaspores. All of the submerged and floating‐leaved plants studied can asexually reproduce via shoot fragments, which are frequently produced by mechanical forces and transported by water flow although *N. peltata* has less breakable stems than the other four species. The emergent *T. latifolia* reproduces vegetatively by rhizomes rather than shoot fragments.

The pollination mode of the two floating‐leaved species, with relatively conspicuous flowers, is entomophilous. Submerged *C. demersum* is hydrophilous and inefficient in pollen dispersal via the aquatic medium. It has a low seed production (Les, 1988; Triest, Thi, Thi, Sierens, & Van Geert, 2010) and its fruits have spines up to 14 mm long (Flora of China 2016). Submerged *M. spicatum* and *P. lucens* and emergent *T. latifolia* undergo anemophilous pollination. The fruits of *H. dubia* and *N. peltata* (fruit diameter 8–26 mm) are larger than those of the other four species (fruit diameter 1–5 mm) (Darbyshire & Francis, 2008; Diao, 1990; Flora of China 2016). There is evidence of bird‐dispersed propagules and internal and/or external transport in each of these six species (Catling, Mitrow, Haber, Posluszny, & Charlton, 2003; Green, Jenkins, Bell, Morris, & Kingsford, 2008; Reynolds & Cumming, 2016; Smits, Van Ruremonde, & Van Der Velde, 1989). Additionally, the fruits of *T. latifolia*, maturing in air, are small enough to be dispersed by wind (Green et al., 2008).

### 2.3 Plant sampling

In each lake, we established 7–11 sampling sites using GPS in areas with abundant aquatic plants in summer 2012 and 2013. Pairwise distances between sampling sites were more than 0.3 km. In each site, 3–9 leaf samples from *M. spicatum*,* C. demersum*,* P. lucens*,* H. dubia*,* N. peltata*, and *T. latifolia* were collected if present. The interval between leaf samples was more than 5 m. Leaf samples were immediately put into plastic bags containing desiccant (allochroic silica gel). The sample size and number of sampling sites (subpopulations) were 177 and 31, respectively, in five lakes (populations) for *M. spicatum*; 174 and 30 in six lakes for *C. demersum*; 117 and 20 in four lakes for *P. lucens*; 98 and 16 in three lakes for *H. dubia*; 148 and 23 in five lakes for *N. peltata*; and 101 and 18 in five lakes for *T. latifolia* (Appendix S1).

### 2.4 DNA extraction and PCR amplification

Total genomic DNA was extracted from dry leaf samples following a modified cetyltrimethyl ammonium bromide (CTAB) protocol (Saghai‐Maroof, Soliman, Jorgensen, & Allard, 1984). From 60 ISSR primers published by the University of British Columbia in 2006 (http://www.biotech.ubc.ca/services/naps/primers/Primers.pdf), for each species we selected 9–13 primers amplified polymorphic and reproducible bands (Appendix S2). Amplification of ISSR was carried out in a volume of 25 μl containing a mix of *Taq* polymerase (0.1 U/μl), dATP, dCTP, dGTP, dTTP (0.4 mM each) and buffer (Beijing ComWin Biotech), ISSR primer (Beijing Genomics Institute), and genomic DNA. PCRs were performed in a C1000 Thermal Cycler (Bio‐Rad Laboratories). A denaturation period of 4 min at 94°C was followed by 40 cycles of 30 s at 94°C, 30 s at 52°C and 1 min 30 s at 72°C, and then 10 min at 72°C for final extension. PCR products were separated on 2% (w/v) agarose gels and visualized under UV after staining with ethidium bromide.

### 2.5 Data analysis

PCR amplification products were scored as presence (1) or absence (0) of bands. Nei's unbiased genetic distances (Nei, 1978) among populations and the eigen values of principle coordinate analyses (PCA) across all individuals were calculated using GenAlEx 6.5 (Peakall & Smouse, 2012). The resulting genetic distance matrix was used for a cluster analysis according to the unweighted pair‐group method with arithmetic averages (UPGMA) using NTSYSpc 2.0 (Rohlf, 1998). Scatter diagrams from the principle coordinate analysis were drawn in SigmaPlot 10.0 (Systat Software, Chicago, IL, USA).

Bayesian cluster analyses of population structure were performed using the software STRUCTURE 2.3 (Pritchard, Stephens, & Donnelly, 2000) to determine the number of genetic clusters in each species, using the admixture model with independent allele frequencies. We tested *K* in 10 independent runs from 1 to 6, without using sampling location as a prior to assess convergence of Ln P (D) (10,000 burn‐in and 10,000 Markov chain Monte Carlo replicates in each run). The value of Ln P (D) is the posterior probability of the data for a given *K*. Furthermore, Δ*K* values based on the rate of change in Ln P (D) between successive *K* values were calculated according to Evanno, Regnaut, and Goudet (2005). Then, based on the distribution of Δ*K* as a function of *K*, we identified the correct number of clusters (*K*) that best explain the data.

Total genetic diversity (*H*_T_), mean genetic diversity within lake populations (*H*_S_), Nei's genetic differentiation index among populations (*G*_ST_), and gene flow among populations (*Nm*, the theoretical number of migrants entering every population per generation) were calculated using POPGENE version 1.32 (Yeh, Yang, Boyle, Ye, & Mao, 1999). Analysis of molecular variance (AMOVA) with 1,000 permutations was conducted using ARLEQUIN version 3.5.1.2 (Excoffier, Smouse, & Quattro, 1992) among all samples to partition variation into hierarchical components (among lake populations, among subpopulations, and within subpopulations for each species).

## 3 RESULTS

### 3.1 Population genetic distances of six aquatic plants

**Figure 3:**
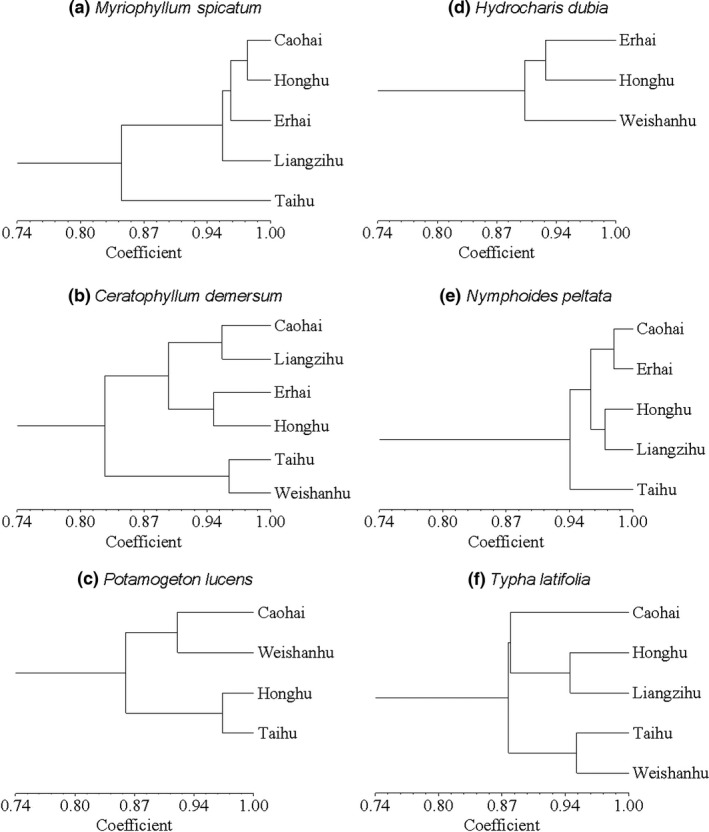
Dendrograms based on analysis of the ISSR binary matrix for lake populations of six aquatic plants using Nei's unbiased genetic identity coefficients (Nei, 1978)

According to UPGMA dendrograms of lake populations based on Nei's unbiased genetic similarity coefficients, populations in Taihu Lake and/or Weishanhu Lake were distant from the other populations for all species except *P. lucens* (Figure 3, Table 2). The Taihu and Weishanhu populations clustered very closely in *C. demersum* and *T. latifolia*. The genetic distance between the two plateau lake populations (Caohai and Erhai) was closer than the distance between other populations only for *N. peltata*. Genetic similarities between populations connected by the Yangtze River were not consistently higher than unconnected populations; however, Honghu Lake and Liangzihu Lake populations had the closest geographic distance among the six lakes and clustered closely in *N. peltata* and *T. latifolia*.

**Table 2:** Pairwise population matrix of Nei's unbiased genetic identity for six aquatic plant species

Species	Genetic identity				
*Myriophyllum spicatum*	Caohai	Erhai	Honghu	Liangzihu	
Erhai	0.964				
Honghu	0.976	0.955			
Liangzihu	0.946	0.941	0.964		
Taihu	0.854	0.820	0.860	0.855	
*Ceratophyllum demersum*	Caohai	Erhai	Honghu	Liangzihu	Taihu
Erhai	0.884				
Honghu	0.927	0.942			
Liangzihu	0.950	0.865	0.906		
Taihu	0.791	0.873	0.848	0.770	
Weishanhu	0.804	0.895	0.868	0.789	0.958
*Potamogeton lucens*	Caohai	Honghu	Taihu		
Honghu	0.824				
Taihu	0.842	0.966			
Weishanhu	0.917	0.888	0.890		
*Hydrocharis dubia*	Erhai	Honghu			
Honghu	0.923				
Weishanhu	0.911	0.890			
*Nymphoides peltata*	Caohai	Erhai	Honghu	Liangzihu	
Erhai	0.981				
Honghu	0.942	0.947			
Liangzihu	0.967	0.973	0.971		
Taihu	0.928	0.941	0.925	0.949	
*Typha latifolia*	Caohai	Honghu	Liangzihu	Taihu	
Honghu	0.887				
Liangzihu	0.871	0.940			
Taihu	0.881	0.886	0.863		
Weishanhu	0.865	0.887	0.878	0.947	

**Figure 4:**
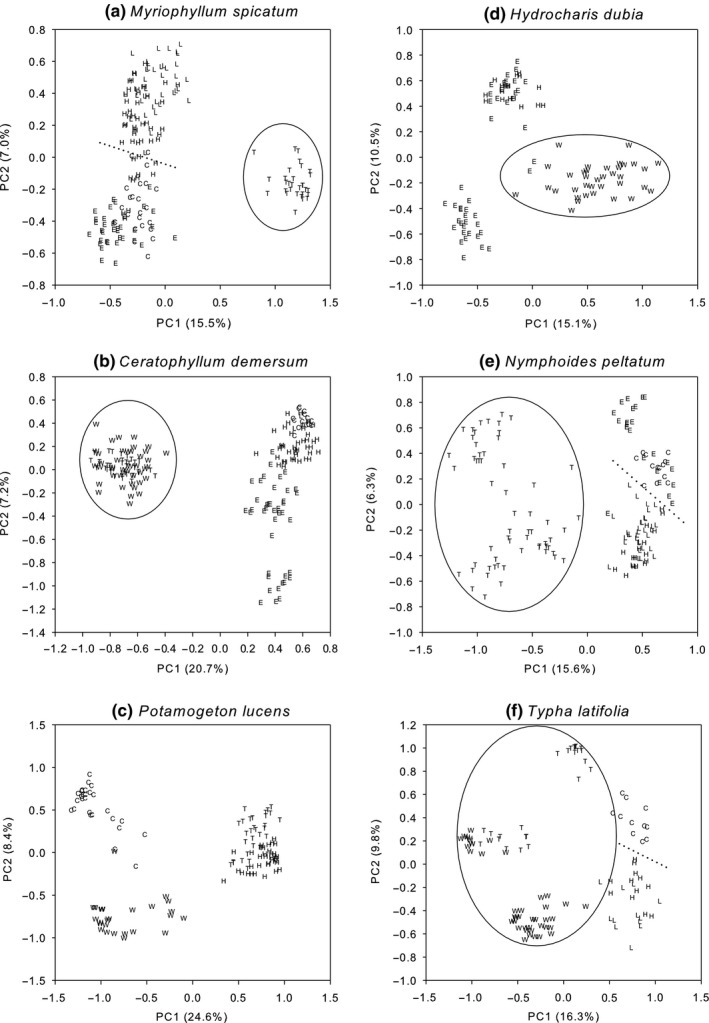
Scatter plots of the first and second principle components based on the analysis of ISSR binary data for individuals of six aquatic species in different lakes. Each letter denotes an individual (C, Caohai Lake; E, Erhai Lake; H, Honghu Lake; L, Liangzihu Lake; T, Taihu Lake; and W, Weishanhu Lake). Ellipses highlight close relationships among individuals collected from Taihu Lake and/or Weishanhu Lake. Dotted lines suggest the tendency for individuals from Caohai and Erhai Lakes to scatter from those from Honghu and Liangzihu Lakes

PCA analyses based on data from individual samples (Figure 4) showed that samples originating from each lake tended to cluster together and this was in agreement with genetic similarity coefficients. Again, the Taihu Lake and/or Weishanhu Lake individuals scattered from all others in all six species except *P. lucens*. In *M. spicatum* and *N. peltata*, except for Taihu Lake individuals, all individuals tended to divide into two groups: one included individuals from the plateau Caohai and Erhai Lakes; and the other included individuals from Honghu and Liangzihu Lakes.

**Figure 5:**
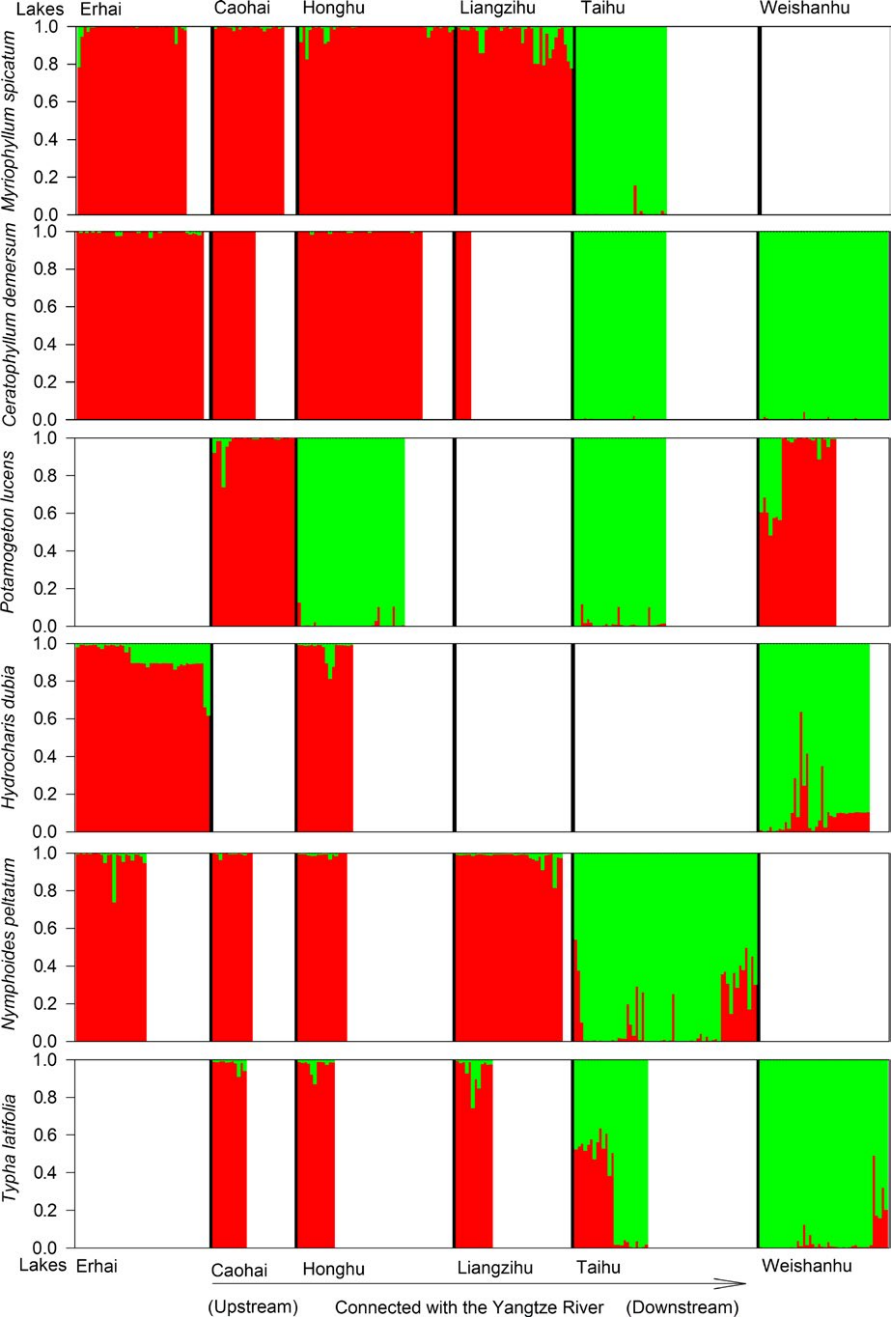
Estimated genetic structure of lake populations of six species of aquatic plants, inferred by a Markov chain Monte Carlo clustering (STRUCTURE) at the individual level (*K* = 2). Black lines indicate different population origins. There are no samples in blank spaces

Bayesian cluster analyses (Figure 5) agreed with the results of UPGMA and PCA analyses that populations of Taihu Lake and/or Weishanhu Lake differentiated from the other lakes for all species except *P. lucens*. In STRUCTURE analysis, the optimal division with each of the six species was found at *K *= 2, as indicated by the data Ln P (D) and the rate of change values (Δ*K*). Thus, the genetic clusters were assigned to two groups. For all species except *P. lucens*, one group included populations of Taihu Lake and/or Weishanhu Lake, the other group including other lakes.

### 3.2 Genetic diversity, differentiation, and gene flow

Total genetic diversity (*H*_T_) and mean within‐population genetic diversity (*H*_S_) varied among the six species (Table 3). The greatest values of *H*_T_ (0.262) and *H*_S_ (0.211) were observed in *H. dubia*; the lowest values of *H*_T_ (0.189) and *H*_S_ (0.154) were found in *N. peltata*. For *C. demersum*, the value of *H*_T_ (0.262) was similar to that of *H. dubia*, but the value of *H*_S_ (0.169) was moderate among the six species.

**Table 3:** Genetic diversity, differentiation, and gene flow among populations of six aquatic plants

Species	Number of lake populations	*H*_T_	*H*_S_	*G*_ST_	*Nm*
*Myriophyllum spicatum*	5	0.243 (0.026)	0.185 (0.015)	0.242	1.569
*Ceratophyllum demersum*	6	0.262 (0.027)	0.169 (0.012)	0.354	0.913
*Potamogeton lucens*	4	0.249 (0.027)	0.178 (0.013)	0.287	1.244
*Hydrocharis dubia*	3	0.262 (0.024)	0.211 (0.016)	0.194	2.084
*Nymphoides peltata*	5	0.189 (0.020)	0.154 (0.012)	0.185	2.204
*Typha latifolia*	5	0.242 (0.031)	0.165 (0.014)	0.320	1.065

*H*_T_, Total genetic diversity; *H*_S_, Mean genetic diversity within lake populations; *G*_ST_, Genetic differentiation index among populations; *Nm,* potential number of migrants per generation.

The values in parentheses are standard deviations.

In terms of values of *G*_ST_ and *Nm* (Table 3) among the six species studied, *H. dubia* (*G*_ST_ = 0.194, *Nm *= 2.084) and *N. peltata* (*G*_ST_ = 0.185, *Nm *= 2.204) had the lowest differentiation and the highest gene flow. *Ceratophyllum demersum* had the greatest genetic differentiation (*G*_ST_ = 0.354) and the lowest gene flow (*Nm *= 0.913). The other three species (*M. spicatum*,* P. lucens*, and *T. latifolia*) had moderate differentiation and gene flow.

AMOVA analyses showed that in the six species 19.29%–36.73% of genetic variation was found among lake populations (Table 4). *Hydrocharis dubia* and *N. peltata* partitioned relatively less genetic variation among populations than the other four species. In within‐population variability, variance among subpopulations was less than that between individuals within subpopulation for each species.

**Table 4:** Analysis of molecular variance (AMOVA)

Source of variation	*df*	SSD	CV	% Total	*p* value
*Myriophyllum spicatum*					
Among lakes (populations)	4	1314.71	8.11	28.09	<.001
Among subpopulations	26	1135.08	4.87	16.87	<.001
Within subpopulations	147	2336.67	15.90	55.04	<.001
Total	177	4786.46	28.88		
*Ceratophyllum demersum*					
Among lakes (populations)	4	1177.06	7.40	32.37	<.001
Among subpopulations	25	1109.49	5.65	20.42	<.001
Within subpopulations	144	1679.58	11.66	47.21	<.001
Total	173	3966.13	24.71		
*Potamogeton lucens*					
Among lakes (populations)	3	1276.37	12.76	36.73	<.001
Among subpopulations	16	859.92	6.58	18.95	<.001
Within subpopulations	97	1493.10	15.39	44.32	<.001
Total	116	3629.39	34.73		
*Hydrocharis dubia*					
Among lakes (populations)	2	334.96	3.90	19.29	<.001
Among subpopulations	13	614.79	6.13	33.38	<.001
Within subpopulations	82	800.50	9.76	47.33	<.001
Total	97	1750.25	19.79		
*Nymphoides peltata*					
Among lakes (populations)	4	1053.24	6.92	20.17	<.001
Among subpopulations	18	1303.45	8.53	24.87	<.001
Within subpopulations	124	2337.72	18.85	54.95	<.001
Total	146	4694.41	34.30		
*Typha latifolia*					
Among lakes (populations)	4	734.20	6.42	23.26	<.001
Among subpopulations	13	838.38	9.45	34.22	<.001
Within subpopulations	83	974.49	11.74	42.52	<.001
Total	100	2547.07	27.61		

*df*, degree of freedom; SSD, sum of squared deviations; CV, variance component estimates; % total, percentage of total variation.

## 4 DISCUSSION

### 4.1 Genetic relationships among lake populations in the six aquatic plants

The plateau lake populations studied here were not consistently genetically distant from plain lake populations. For *N. peltata* and *M. spicatum* only, individuals could be generally divided into plateau (Caohai and Erhai Lakes) and plain (Honghu and Liangzihu Lakes) groups (excluding Taihu Lake individuals) via PCA analysis. Previous studies on these species in China have yielded similar findings (Liao et al., 2013; Wu et al., 2016). Noticeable local differentiation was observed in each of the six species because samples originating from each lake tended to cluster together, like that in *Potamogeton maackianus* in the middle reaches of the Yangtze River (Li, Xia, Li, & Wang, 2004) and the seagrass *Halodule wrightii* from Christmas Bay (Angel, 2002). Local differentiation may attribute to divergent environmental pressures and demographic factors such as population size and lake isolation (Li et al., 2004; Liao et al., 2013; Wang & Bradburd, 2014). Hydrologic connectivity seems not appear to significantly contribute to genetic similarities between lakes because genetic distances were not consistently closer between Yangtze River‐connected lakes than between unconnected lakes.

Despite belonging to different river systems and climates, the Taihu Lake and Weishanhu Lake populations clustered very closely for *C. demersum* and *T. latifolia*. One explanation may be bird‐mediated dispersal. Taihu and Weishanhu Lakes are important stopover sites for shorebirds along the East Asian‐Australasian flyway and are extensively used by waterfowl (Li & Yang, 1997). Many authors have argued that compelling evidence is provided by existing plant distributions that match migration routes (Santamaría, 2002).

Surprisingly, the Taihu and/or Weishanhu populations were genetically distant from other populations in all species except *P. lucens*, which seems difficult to explain. For Taihu Lake, perhaps its lagoon origin, salinity and repeated sea–land alternation (Wang & Dou, 1998) have acted in selective ways to affect the adaptations of plants inhabiting this system. Alternatively, as Chen et al. (2009) speculate, Taihu Lake is the only lake to not flood during three catastrophic floods in the past 100 years, resulting in a lack of propagule dispersal and distant genetic relationships between Taihu Lake and other Yangtze River‐connected lakes. However, the explanation provided by Chen et al. (2009) is not applicable to our study because not all lakes studied are in the Yangtze River system. It is puzzling that the Weishanhu population was distant from other populations except for Taihu. Weishanhu Lake is a freshwater lake formed as a result of depositing sediment carried by rivers (Wang & Dou, 1998). A clue to this puzzle might be that Weishanhu Lake is the only lake in the North China Plain, with a warm temperate monsoon climate and in the Huaihe River system. The Honghu and Liangzihu populations, with the closest geographic distance in this study, had a close genetic relationship for *N. peltata*,* T. latifolia*, and *M. spicatum*. This high similarity may be the result of multiple evolutionary forces including similar climate, hydrologic connectivity, and bird‐mediated dispersal (Chen et al., 2007, 2009).

### 4.2 Genetic diversity, differentiation, and gene flow in the six species

The values of within‐population genetic diversity (*H*_S_ = 0.154–0.211) of the six species found in the present work were within the range of the reported values of plant genetic diversity and similar to those of the widespread aquatic species (Chen, Li, Yin, & Li, 2008; Chen et al., 2007, 2009; Liao et al., 2013; Nybom, 2004; Wang et al., 2010). The genetic diversity of *N. peltata* measured in this study was considerably lower than that found by Liao et al. (2013), which may be related to the differences in molecular markers and sample size between the two studies.

High genetic differentiation commonly indicates low gene flow among populations, as was the case in *C. demersum*, while the opposite was true for *H. dubia* and *N. peltata*. Although gene flow can be realized through pollen and propagule flow, the primary contributor of gene flow between geographically distant populations should be propagules rather than pollens (Chen et al., 2009) due to distance limitation of pollens being transported by water, wind, and insects (Tero, Aspi, Siikamäki, Jäkäläniemi, & Tuomi, 2003). Propagule flow can be mediated by water, aquatic animals such as fish, wind, and birds (Li, 2014). At a large geographic scale, however, only bird‐mediated dispersal may be largely responsible for the two‐dimensional dispersal patterns measured here, such as is the case in *P. malaianus* (Chen et al., 2009). Low gene flow among hydrophilous *C. demersum* populations may be explained primarily by low seed production (Les, 1988; Triest et al., 2010) and the long fruit spines which possibly discourage bird ingestion. In contrast, the relatively high gene flow of entomophilous *H. dubia* and *N. peltata* may be related to their large and edible fruits (Flora of China 2016).

Overall, for sympatric and widespread aquatic species with different biological traits, there may be similar aspects to population genetic structure over a large geographic scale. Differences in environmental factors between plateau and plain lakes and long distance hydrochory have limited importance on aquatic plant genetic structures. However, gene flow mediated by birds may play the most important role in the formation of genetic patterns. The close genetic relationship between Taihu Lake and Weishanhu Lake populations, each in different river systems and with different climates, may be related to the migration routes of birds. Differences in gene flow among the six aquatic plants may be attributable to different bird‐transport and the fruit traits of each species.

## CONFLICT OF INTEREST

None declared.
