# Supplementary material for: Population genetic structure in six sympatric and widespread aquatic plants inhabiting diverse lake environments in China
Source: Ecol Evol. 2017 Jun 15;7(15):5713–23. doi: 10.1002/ece3.3141 (PMC5552939; doi:10.1002/ece3.3141)
Supplement: Supplementary file 1 [file ECE3-7-5713-s001.doc]

**Supporting information**

**Appendix S1** Sample sizes of six aquatic plant species

| Species/ Lake (population) | Number of subpopulations | Sample size | Total bands | Bands per primer |
| --- | --- | --- | --- | --- |
| *Myriophyllum spicatum* (12 primers) | | | |  |
| Caohai | 5 | 23 | 129 | 11 |
| Erhai | 7 | 36 | 142 | 12 |
| Honghu | 8 | 50 | 141 | 12 |
| Liangzihu | 6 | 38 | 141 | 12 |
| Taihu | 5 | 30 | 131 | 11 |
| Total | 31 | 177 | 185 | 15 |
|  |  |  |  |  |
| *Ceratophyllum demersum* (9 primers) | | | |  |
| Caohai | 3 | 14 | 71 | 8 |
| Erhai | 7 | 42 | 105 | 12 |
| Honghu | 7 | 41 | 97 | 11 |
| Liangzihu | 1 | 5 | 48 | 5 |
| Taihu | 5 | 30 | 102 | 11 |
| Weishanhu | 7 | 42 | 106 | 12 |
| Total | 30 | 174 | 139 | 15 |
|  |  |  |  |  |
| *Potamogeton lucens* (11 primers) | | | |  |
| Caohai | 5 | 27 | 146 | 13 |
| Honghu | 6 | 35 | 155 | 14 |
| Taihu | 5 | 30 | 163 | 15 |
| Weishanhu | 4 | 25 | 135 | 12 |
| Total | 20 | 117 | 202 | 18 |
|  |  |  |  |  |
| *Hydrocharis dubia* (9 primers) | | | |  |
| Erhai | 7 | 44 | 98 | 11 |
| Honghu | 3 | 18 | 83 | 9 |
| Weishanhu | 6 | 36 | 97 | 11 |
| Total | 16 | 98 | 115 | 13 |
|  |  |  |  |  |
| *Nymphoides peltata* (11 primers) | | | |  |
| Caohai | 2 | 13 | 102 | 9 |
| Erhai | 4 | 24 | 154 | 14 |
| Honghu | 2 | 16 | 111 | 10 |
| Liangzihu | 6 | 35 | 161 | 15 |
| Taihu | 9 | 60 | 191 | 17 |
| Total | 23 | 148 | 219 | 20 |
|  |  |  |  |  |
| *Typha latifolia* (13 primers) | | |  |  |
| Caohai | 2 | 11 | 147 | 11 |
| Honghu | 2 | 12 | 158 | 12 |
| Liangzihu | 3 | 12 | 162 | 12 |
| Taihu | 4 | 24 | 176 | 14 |
| Weishanhu | 7 | 42 | 167 | 13 |
| Total | 18 | 101 | 204 | 16 |

**Appendix S2** ISSR primers used in this study

| Primer | Sequence (5’-3’) | Total bands | PPB (%) |
| --- | --- | --- | --- |
| *Myriophyllum spicatum* (12 primers) | |  |  |
| UBC808 | AGAGAGAGAGAGAGAGC | 18 | 100 |
| UBC825 | ACACACACACACACACT | 14 | 100 |
| UBC826 | ACACACACACACACACC | 19 | 100 |
| UBC836 | AGAGAGAGAGAGAGAGTA | 16 | 100 |
| UBC840 | GAGAGAGAGAGAGAGATT | 16 | 100 |
| UBC841 | GAGAGAGAGAGAGAGACC | 19 | 100 |
| UBC855 | ACACACACACACACACTT | 18 | 100 |
| UBC856 | ACACACACACACACACCA | 15 | 100 |
| UBC864 | ATGATGATGATGATGATG | 10 | 100 |
| UBC888 | CACCACACACACACACA | 12 | 100 |
| ISSR8^†^ | CACACACACACACAATCC | 14 | 100 |
| ISSR19^†^ | GTGCACACACACACACA | 14 | 100 |
|  |  |  |  |
| *Ceratophyllum demersum* (9 primers) | |  |  |
| UBC807 | AGAGAGAGAGAGAGAGT | 17 | 100 |
| UBC810 | GAGAGAGAGAGAGAGAT | 14 | 100 |
| UBC825 | ACACACACACACACACT | 17 | 100 |
| UBC826 | ACACACACACACACACC | 18 | 100 |
| UBC836 | AGAGAGAGAGAGAGAGTA | 16 | 100 |
| UBC840 | GAGAGAGAGAGAGAGATT | 18 | 100 |
| UBC842 | GAGAGAGAGAGAGAGATG | 15 | 100 |
| UBC855 | ACACACACACACACACTT | 14 | 100 |
| UBC866 | CTCCTCCTCCTCCTCCTC | 10 | 100 |
|  |  |  |  |
| *Potamogeton lucens* (11 primers) | |  |  |
| UBC807 | AGAGAGAGAGAGAGAGT | 13 | 100 |
| UBC809 | AGAGAGAGAGAGAGAGG | 20 | 100 |
| UBC810 | GAGAGAGAGAGAGAGAT | 18 | 100 |
| UBC840 | GAGAGAGAGAGAGAGAYT | 20 | 100 |
| UBC842 | GAGAGAGAGAGAGAGAYG | 18 | 100 |
| UBC844 | CTCTCTCTCTCTCTCTRC | 16 | 100 |
| UBC845 | CTCTCTCTCTCTCTCTRG | 17 | 100 |
| UBC880 | GGAGAGGAGAGGAGA | 19 | 100 |
| UBC881 | GGGTGGGGTGGGGTG | 20 | 100 |
| UBC886 | VDVCTCTCTCTCTCTCT | 21 | 100 |
| UBC890 | VHVGTGTGTGTGTGTGT | 20 | 100 |
|  |  |  |  |
| *Hydrocharis dubia* (9 primers) | |  |  |
| UBC808 | AGAGAGAGAGAGAGAGC | 17 | 100 |
| UBC810 | GAGAGAGAGAGAGAGAT | 17 | 100 |
| UBC811 | GAGAGAGAGAGAGAGAC | 10 | 100 |
| UBC840 | GAGAGAGAGAGAGAGAYT | 14 | 100 |
| UBC841 | GAGAGAGAGAGAGAGACC | 10 | 100 |
| UBC842 | GAGAGAGAGAGAGAGAYG | 11 | 100 |
| UBC855 | ACACACACACACACACTT | 11 | 100 |
| UBC864 | ATGATGATGATGATGATG | 10 | 90 |
| UBC899 | CATGGTGTTGGTCATTGTTCCA | 15 | 100 |
|  |  |  |  |
| *Nymphoides peltata* (11 primers) | |  |  |
| UBC807 | AGAGAGAGAGAGAGAGT | 21 | 100 |
| UBC808 | AGAGAGAGAGAGAGAGC | 17 | 100 |
| UBC809 | AGAGAGAGAGAGAGAGG | 19 | 100 |
| UBC815 | CTCTCTCTCTCTCTCTG | 19 | 100 |
| UBC825 | ACACACACACACACACT | 15 | 100 |
| UBC826 | ACACACACACACACACC | 16 | 100 |
| UBC835 | AGAGAGAGAGAGAGAGYC | 20 | 100 |
| UBC836 | AGAGAGAGAGAGAGAGYA | 19 | 100 |
| UBC844 | CTCTCTCTCTCTCTCTRC | 22 | 100 |
| UBC845 | CTCTCTCTCTCTCTCTRG | 26 | 100 |
| UBC850 | GTGTGTGTGTGTGTGTYC | 25 | 100 |
|  |  |  |  |
| *Typha latifolia* (13 primers) | |  |  |
| UBC810 | GAGAGAGAGAGAGAGAT | 14 | 93 |
| UBC823 | TCTCTCTCTCTCTCTCC | 13 | 85 |
| UBC827 | ACACACACACACACACG | 15 | 100 |
| UBC834 | AGAGAGAGAGAGAGAGYT | 12 | 100 |
| UBC835 | AGAGAGAGAGAGAGAGYC | 20 | 100 |
| UBC836 | AGAGAGAGAGAGAGAGYA | 12 | 67 |
| UBC840 | GAGAGAGAGAGAGAGAYT | 12 | 83 |
| UBC842 | GAGAGAGAGAGAGAGAYG | 16 | 81 |
| UBC845 | CTCTCTCTCTCTCTCTRG | 18 | 94 |
| UBC855 | ACACACACACACACACYT | 20 | 100 |
| UBC873 | GACAGACAGACAGACA | 18 | 100 |
| UBC881 | GGGTGGGGTGGGGTG | 17 | 88 |
| UBC888 | BDBCACACACACACACA | 17 | 71 |

*PPB, percentage of polymorphic bands. ^†^The two primers are referred from the literature (Triest et al. 2010). Y = (C, T), R = (A, G), V = (A, C, G) , H = (A, C, T).


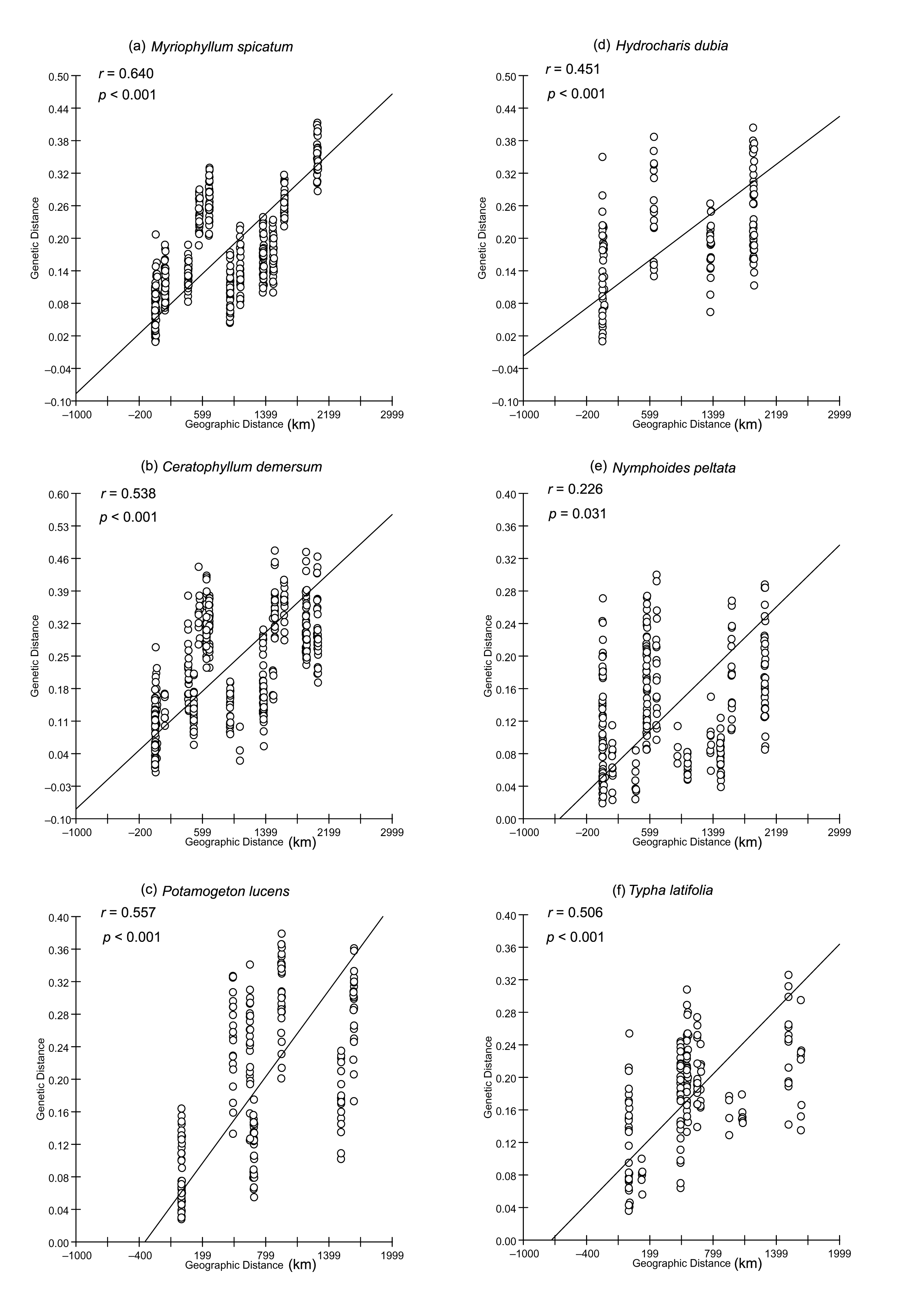


**Figure as Supporting information**

Relationships between pairwise Nei’s unbiased genetic distances and geographic distances calculated via Latitude/ Longitude coordinators between all subpopulations (sampling sites) of each aquatic species. Mantel tests were performed using Isolation By Distance Web Service Version 3.23 (http://ibdws.sdsu.edu/~ibdws/, May 5, 2017; Bohonak, A. J. 2002. IBD (Isolation by Distance): a program for analyses of isolation by distance. J. Hered. 93: 153–154).
